# KANK1-NTRK3 fusions define a subset of BRAF mutation negative renal metanephric adenomas

**DOI:** 10.1186/s12881-020-01143-6

**Published:** 2020-10-12

**Authors:** Aida Catic, Amina Kurtovic-Kozaric, Ardis Sophian, Lech Mazur, Faruk Skenderi, Ondrej Hes, Stephen Rohan, Dinesh Rakheja, Jillene Kogan, Michael R. Pins

**Affiliations:** 1 Department of Cytogenetics, ACL Laboratories, Rosemont, IL USA; 2 Department of Genetics and Bioengineering, International Burch University, Francuske revolucije bb, Ilidza, 71000 Sarajevo, Bosnia and Herzegovina; 3 Department of Clinical Pathology, Cytology and Human Genetics, Clinical Center of the University of Sarajevo, Sarajevo, Bosnia and Herzegovina; 4 Department of Pathology, Charles University Hospital Pilsen, Pilsen, Czech Republic; 5 Department of Pathology, Saint Joseph Hospital, Denver, CO USA; 6 Department of Pathology and Pediatrics, University of Texas Southwestern Medical Center, Dallas, TX USA; 7 Departments of Pathology and Laboratory Medicine, Children’s Health, Dallas, TX USA; 8 Department of Pathology, Advocate Lutheran General Hospital, Park Ridge, IL USA; 9 Department of Pathology, Chicago Medical School of Rosalind Franklin University of Medicine and Science, North Chicago, IL USA; 10 Advocate Medical Group Genetics, Park Ridge, IL USA

**Keywords:** Metanephric adenoma, Cytogenetics, Chromosomal translocations, *KANK1-NTRK3* fusion, *BRAF*^V600E^

## Abstract

**Background:**

Metanephric adenoma (MA) is a rare benign renal neoplasm. On occasion, MA can be difficult to differentiate from renal malignancies such as papillary renal cell carcinoma in adults and Wilms̕ tumor in children. Despite recent advancements in tumor genomics, there is limited data available regarding the genetic alterations characteristic of MA. The purpose of this study is to determine the frequency of metanephric adenoma cases exhibiting cytogenetic aberration t (9;15)(p24;q24), and to investigate the association between t (9,15) and *BRAF* mutation in metanephric adenoma.

**Methods:**

This study was conducted on 28 archival formalin fixed paraffin-embedded (FFPE) specimens from patients with pathologically confirmed MA. Tissue blocks were selected for *BRAF* sequencing and fluorescent in situ hybridization (FISH) analysis for chromosomal rearrangement between *KANK1* on chromosome 9 (9p24.3) and *NTRK3* on chromosome 15 (15q25.3), which was previously characterized and described in two MA cases.

**Results:**

*BRAF*^V600E^ mutation was identified in 62% of our cases, 9 (38%) cases were *BRAF*^WT^, and 4 cases were uninformative. Of the 20 tumors with FISH results, two (10%) were positive for *KANK1-NTRK3* fusion. Both cases were *BRAF*^WT^ suggesting mutual exclusivity of *BRAF*^V600E^ and *KANK1-NTRK3* fusion, the first such observation in the literature.

**Conclusions:**

Our data shows that *BRAF* mutation in MA may not be as frequent as suggested in the literature and *KANK-NTRK3* fusions may account for a subset of *BRAF*^WT^ cases in younger patients. FISH analysis for *KANK1-NTRK3* fusion or conventional cytogenetic analysis may be warranted to establish the diagnosis of MA in morphologically and immunohistochemically ambiguous MA cases lacking *BRAF* mutations.

## Background

Metanephric adenoma (MA) is a rare benign renal tumor classified under the rubric of metanephric tumors, which also include metanephric stromal tumor and metanephric adenofibroma [1]. *BRAF* mutations have been identified in metanephric stromal tumor and metanephric adenofibroma in addition to metanephric adenoma, which justifies their grouping as family of metanephric tumors by the World Health Organization (WHO) [2]. MA is uncommon and generally occurs in adults between the fourth and sixth decades of life and occasionally in children [3]. The male-to-female ratio is between 1:2 to 1:3 with a mean age of approximately 41 years [3, 4]. In adults, MA accounts for approximately 0.2% of adult renal epithelial neoplasms [5]. Despite the fact that fewer than 25 cases have been reported in children, it is considered to be the most common benign pediatric renal epithelial tumor [6, 7]. Including both pediatric and adult cases, fewer than 200 cases of MA have been reported in the literature, thus, illustrating its rarity and the scarcity of available data [5–8].

The majority of MA cases can be diagnosed on routine hematoxylin and eosin (H&E) stained slides. However, in some challenging cases MA can be difficult to morphologically differentiate from malignant renal neoplasms [9]; particularly the solid variant of papillary renal cell carcinoma (PRCC) and epithelial-predominant Wilms’ tumor (WT) [10]. The distinction between these renal tumor subtypes can be aided by the use of diagnostic modalities such as immunohistochemistry, cytogenetic studies, and advanced molecular analyses [3]. The correct classification of a renal tumor is not only critical from a diagnostic standpoint, but also from a prognostic and therapeutic standpoint [11, 12].

Immunohistochemistry may be helpful in distinguishing metanephric adenoma from solid variant of papillary renal cell carcinoma (s-pRCC) and epithelial predominant Wilm’s tumor (e-WT). Specifically, MAs are generally diffusely CD57 and WT1 positive, only focally CK7 positive, and CD56 and AMACR negative (rarely weakly positive), s-pRCCs are generally diffusely CK7 and AMACR positive, often CD57 positive (in contrast to conventional [non solid-variant] pRCC, which is usually CD57 negative) and WT1 and CD56 negative, and e-WTs are generally diffusely CD56 and WT1 positive and variably CD57, CK7, and AMACR positive.

The genetic alterations underlying MA tumorigenesis have only been defined relatively recently [11]. Previous cytogenetic studies have revealed a paucity of genetic alterations in MA. The molecular and cytogenetics data reported in the literature in regard to MA is sparse and often consists of literature reviews of previously published cases or isolated case reports. We and another group each reported a case of MA showing a t (9;15) [11–13]. A study by Choueiri et al. demonstrated that approximately 90% of MAs harbor *BRAF*^V600E^ mutations; the genetics of the remaining 10% in their study is unclear [14]. *BRAF*^V600E^ gene mutations are frequently detected in a wide range of benign and malignant human tumors, however, *BRAF* mutations in renal tumors such as renal cell carcinoma (RCC), oncocytoma, and WT are essentially absent [4, 15–21]. This data coupled with Choueiri’s data suggests that *BRAF* mutations are specific for MA amongst renal tumors.

The present study was undertaken to determine the frequency of MA cases exhibiting cytogenetic aberration t (9;15)(p24;q24) as previously reported in the literature, and to investigate the association between t (9;15) and *BRAF* mutation in MAs [11–13]. Two cases included in this study have previously been reported in literature (case # 1 and #12-Table 1.). Catic et al. have demonstrated the specific gene fusion that results from the chromosomal translocation t (9,15)(p24;q24) [11]. Rakheja et al. reported chromosomal translocation mentioned above with only a karyotype [13]. We examined 28 cases of MA at the genetic and molecular level, using a combination of *BRAF* sequencing and fluorescent in situ hybridization (FISH) to detect chromosomal rearrangement between *KANK1* on chromosome 9 (9p24.3) and *NTRK3* on chromosome 15 (15q25.3).

**Table 1:** Clinical, pathological, cytogenetic, and molecular findings^a^

*Case no.*	*Age range years*	*Gender*	*Tumor Size (cm)*	*Laterality*	*Karyotype*	*BRAF Status*	*t(9;15)(p24;q24) FISH*
1.	21–30	F	2	Right	46,XX,t(9;15)(p24;q24)	Wild type	Fusion Present
2.	21–30	F	3	Left	46,XX,t(6;22)(q26;q11.2)	Wild type	Normal
3.	41–50	M	12	NA	46,XY	V600E Mutation	Normal
4.	51–60	F	1.4	Left	46,XX	V600E Mutation	Normal
5.	51–60	M	3	Right	46,XY	V600E Mutation	Normal
6.	31–40	F	6.7	Left	NA	V600E Mutation	Normal
7.	61–70	F	NA	Right	NA	Uninformative	Uninformative
8.	51–60	M	NA	NA	NA	V600E Mutation	Uninformative
9.	51–60	F	0.5	Right	NA	Uninformative	Uninformative
10.	61–70	F	5.1	Right	NA	V600E Mutation	Normal
11.	31–40	F	3.4	Left	NA	V600E Mutation	Normal
12.	1–10	M	1.7	Right	46,XY,t(9;15)(p24;q24),inv.(12)(q13q15)	Wild type	Fusion Present
13.	1–10	M	1.5	Right	NA	V600E Mutation	Normal
14.	31–40	M	4.3	NA	NA	V600E Mutation	Normal
15.	11–20	M	7	Right	NA	Wild type	Normal
16.	NA	NA	NA	NA	NA	Wild type	Normal
17.	51–60	F	NA	Left	NA	Wild type	Normal
18.	51–60	F	5	NA	NA	Uninformative	Uninformative
19.	71–80	M	1.8	Right	NA	Wild type	Normal
20	21–30	F	2	NA	NA	Wild type	Uninformative
21.	61–70	F	4	NA	NA	Wild type	Uninformative
22.	71–80	F	2.5	NA	NA	V600E Mutation	Normal
23.	51–60	F	5	NA	NA	Uninformative	Uninformative
24.	51–60	F	3	NA	NA	V600E Mutation	Normal
25.	11–20	F	3	Right	NA	V600E Mutation	Normal
26.	41–50	F	1.5	NA	NA	V600E Mutation	Uninformative
27.	61–70	M	8	NA	NA	V600E Mutation	Normal
28.	61–70	M	3	NA	NA	V600E Mutation	Normal

^a^*Abbreviations*: *NA* indicates not available, *FISH* Fluorescent in situ hybridization

## Methods

### Patients and samples

This study was conducted on 28 archival formalin fixed paraffin-embedded (FFPE) specimens from renal metanephric adenomas. FFPE blocks and H&E stained slides were obtained from the departments of pathology at four participating institutions, including Advocate Lutheran General Hospital (Park Ridge, IL, USA), Northwestern Memorial Hospital (Chicago, IL, USA), Children’s Medical Center of Dallas (Dallas, TX, USA), and Charles University Hospital (Plzen, Czech Republic). Fourteen cases were of American origin and 14 of European origin. All samples received for this study and data reported have been de-identified. Because this was a retrospective study, ethics committee ruled that no formal ethics approval was required in this particular study.

**Fig. 1:**
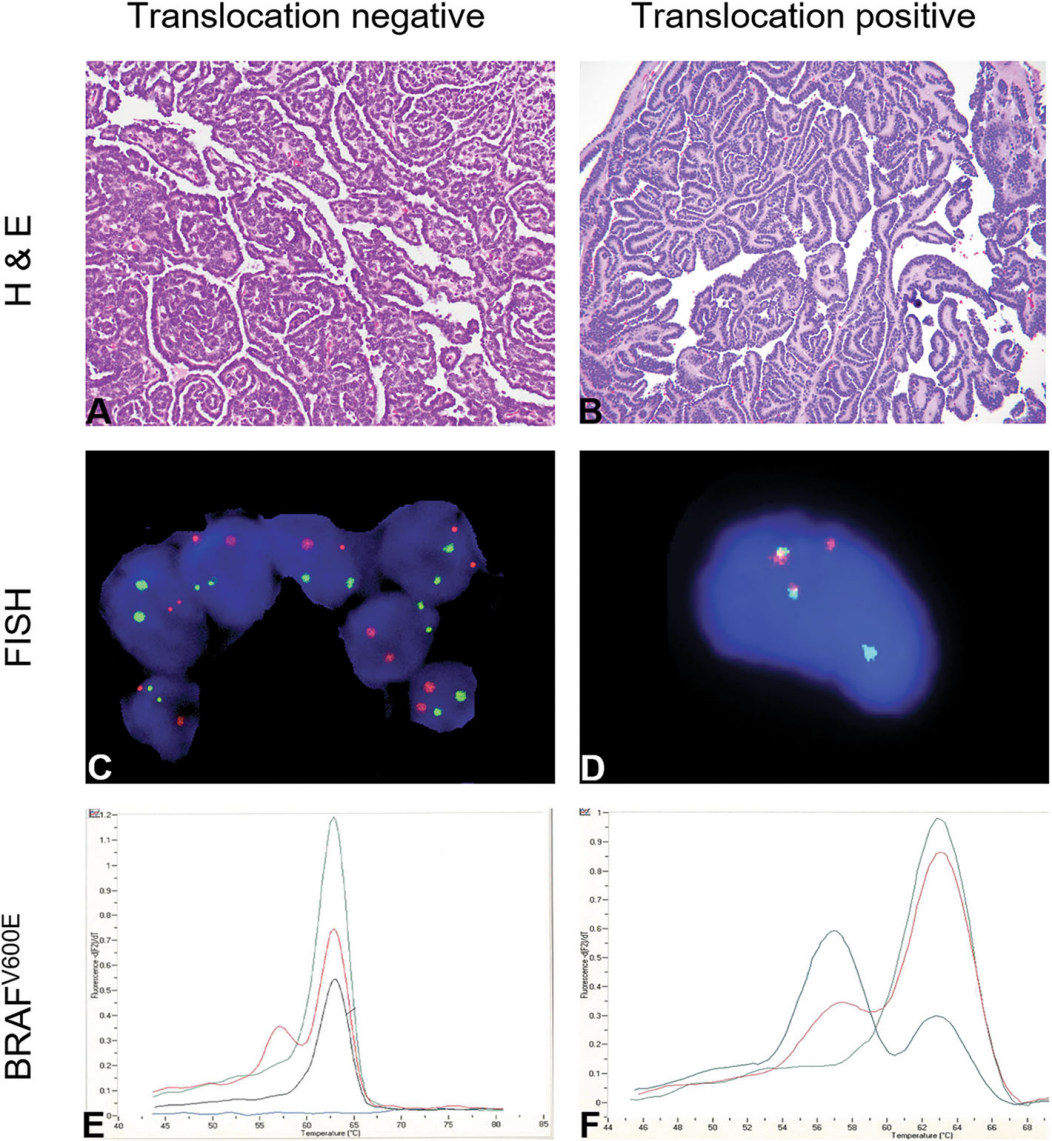
Histologic, immunohistochemical, fluorescence in situ hybridization and molecular genetic analyses findings of metanephric adenoma. **a** Hematoxylin and eosin (H&E) of translocation negative metanephric adenoma case, 200x total magnification **b** H&E slide of translocation positive metanephric adenoma case, 200x total magnification **c** Fluorescence in situ hybridization with probes for chromosomes 9 (green signal) and 15 (orange/red signal) show normal nuclei with two red and two green signals, (original magnifications 100x) **d** Abnormal fluorescence in situ hybridization utilizing 2 color probes for the t(9;15) showing one green (9p24) and one orange/red (15q24) signal on the normal homologues. The yellow signals represent the fusion of the probes on the abnormal homologues of chromosome 9p24 and 15q24 (original magnifications 100x) **e** Melting curve analysis of *BRAF* mutations in metanephric adenoma sample illustrating a sample without a *BRAF* GTG > GAC (V600E) mutation of codon 600 (nucleotide 1799) **f**. Melting curve profiles illustrating the detection of a *BRAF*^V600E^ mutation of the metanephric adenoma sample with a GTG > GAC (V600E) mutation of codon 600 (nucleotide 1799). Green melting curve represents quality control (QC) wild-type (WT), red melting curve represents QC positive for V600E, black curve represents patient samples (E-non-mutated; F-BRAF^V600E^ mutated) are compared, additional melting peaks or changes in peak-area ratios indicate a sequence alteration under the probe (E and F)

**Fig. 2:**
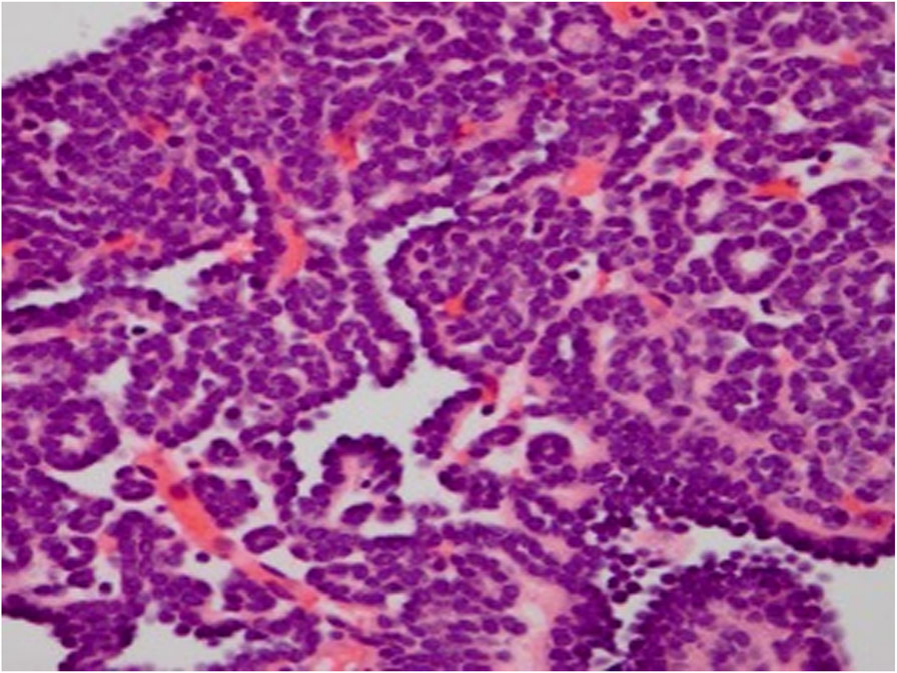
Hematoxylin and eosin photomicrograph of *BRAF* wild type case, showing classic features of renal metanephric adenoma. Small cells with a high nuclear to cytoplasmic ratio form small tubules, rudimentary intraluminal buds and sheets

The MA specimens and hematoxylin and eosin-stained slides were retrieved and reviewed by expert pathologists at each institution. All pathologic specimens were acquired after partial or complete nephrectomy, and none were diagnosed by needle biopsy. The diagnosis of MA was then re-confirmed by two genitourinary pathologists (Fig. 1) and de-identified representative tissue blocks were further selected for *BRAF*^V600E^ exon 15 sequencing and FISH analysis. Histologically, the tumors are composed of epithelial cells arranged in tubules and papillary configurations. The relatively small tumor cells have a high nuclear: cytoplasmic ratio, ovoid nuclei, uniformly dispersed chromatin, inconspicuous nucleoli, scant eosinophilic cytoplasm, and ill-defined cell borders with nuclear overlap, Fig. 1, a and b. Mitoses are not conspicuous. Occasional psammomatoid calcifications are seen, Fig. 2. Patient demographics and clinicopathologic characteristics such as: age, gender, tumor size, laterality, and chromosomal analysis results were provided by pathologists at each institution (Table 1).

### Fluorescence in situ hybridization (FISH)

Metaphase chromosome spreads and interphase nuclei were prepared on a glass microscope slide in accordance with standard cytogenetic procedure and according to the manufacturer’s instructions. Paraffin embedded tissue slides were cut at 2-μm thickness using a microtome and floated in a protein free waterbath at 40 °C. A concurrent H&E slide was stained and marked by a pathologist to delineate the area of tumor for analysis. Fluorescent in situ hybridization probes were purchased from BlueGnome (Illumina, Cambridge, United Kingdom) and Empire Genomics (Buffalo, NY, USA). Briefly, prepared slides from the tumor were placed in a Coplin jar with 40 mL of 2XSSC pH 7.0 at 37 °C for 15 min. Treatment of slides in 2XSSC was used to artificially age the chromosomes, making them less sensitive to over-denaturation. Next, the slides were dehydrated in 70, 85, and 100% ethanol at room temperature for 2 min each, followed by drying on a 50 °C warmer for 15 min. Paraffin embedded tissue slides were baked in a 60 °C oven for a minimum of 1 h. These slides were then placed on the VP processor (Abbott Molecular, Des Plaines, IL, USA) for de-paraffinization, pretreatment, and protease digestion. A mixture of 3.5 μl of locus specific identifier (LSI) hybridization buffer (Abbott Molecular, Des Plaines, IL, USA), 1 μl of sterile water, and 0.5 μl of probe was prepared for the BlueGnome probes and 4 μl of Empire Genomics buffer was used with 1 μl of probe for the Empire probes for each hybridization area. 5 μl of probe mixture was applied to each hybridization area of patient and control slides. Prepared slides and probes were then co-denatured using the ThermoBrite machine at a denature temperature of 76 °C for 5 min and then hybridized overnight. After hybridization, slides were washed using 40 ml of 0.4X SSC/0.3%NP40 for 2 min, followed by 40 ml of 2X SSC/0.1%NP40 for 1 min to remove any excess or unbound probe. After slides were air-dried, 10 μl of DAPI II counterstain on a 22 × 22 coverslip was applied to the targeted area of the slide.

FISH analysis was performed following standard techniques using a fluorescent microscope with appropriate filters (Olympus, Tokyo, Japan). The number of hybridization signals for each probe was assessed on a minimum of twenty metaphases from fresh tumor slides and from 200 nuclei on the paraffin embedded tissue slides with strong and well-delineated signals, and were further selected for thorough examination using Applied Spectral Imaging (ASI) software (Carlsbad, CA, USA). Karyotypes were described and reported in accordance with the International System Committee for Human Cytogenomic Nomenclature (ISCN) 2016 [22].

Probes used to interrogate the **9q24** region were: RP11-143 M15 (9p24.3-green), RP11-59O6 (9p24.3-orange), RP11-130C19 (9p24.3-green), and RP11-1107A23 (9p24.3-green). Probes used to interrogate the **15q24** region were: RP11-62D2 (15q25.3-orange) and RP11-608H9 (15q25.3-orange).

### *BRAF* mutation analysis

FFPE sections were evaluated for the *BRAF* mutation on a Roche LightCycler 2.0 instrument (Mannheim, Germany) utilizing the allelic discrimination by real time polymerase chain reaction (PCR), which was performed at the Clinical Laboratory Improvements Amendments (CLIA) certified ACL Laboratories (Rosemont, IL, USA). In short, the appropriate FFPE tissue block was selected by the pathologist. Four-micrometer-thick sections from FFPE tissue blocks were enriched by manual micro-dissection and DNA was isolated by the ZymoPinPiont method (Irvine, CA, USA), according to the manufacturer’s protocol. Exon 15 of the *BRAF* gene was amplified from 50 ng of genomic DNA by real time PCR using sequence specific primers ordered from Invitrogen-Life Technologies (Carlsbad, CA, USA) (forward primer: 5′-CTCTTCATAATGCTTGCTCTGATAGG-3′, and reverse primer: 5′-TAGTAACTCAGCAGCATCTCAGG-3′). Melting curve analysis was performed by optimized fluorescent probes 5′-FL-TGGAGTGGGTCCCATCAGTTTGAACAGTTGTCTGGATCCATT SpacerC35’-TGGTCTAGCTACAGTGAAATCTC-LC640. The PCR products were amplified in the following conditions: initial denaturation at 95 °C for 10 min; amplification 45 cycles of 95 °C for 5 s, 60 °C for 10 s, and 72 °C for 20 s; melting curves 1 cycle of 95 °C for 2 min, 40 °C for 2 min, and 85 °C at 5 s; and cooling period of 1 cycle at 40 °C for 30 s. The method of *BRAF* exon 15-mutation analysis interpretation has been previously described [4, 11, 14]. Specifically, our assay detects 28 nucleotide changes involving the following codons: V600E, M, L, R, Q, D, K, A and G, L597V, S, Q, R and L, K601E, del, and N, A598V, A598_T599insV, T599I, T599_V600insT, T599_V600insTT, and V600_K601 > E.

Fisher’s exact analysis was performed for comparison between the differential prevalence of BRAF^V600E^ mutation in patients under the age of 30 and those patients over 30 years of age. A *p* value <.05 was used to indicate statistical significance.

## Results

We analyzed 28 MAs. Among those, there were 17 women, 10 men and 1 unknown (F:M, 1.7:1). Patient age ranged from 9.8 to 73 years with median age of 52.5 years (52 among women; and 53.5 among men). Twenty patients were over the age of 30, while 7 were under the age of 30 and 1 patient was of unknown age (Table 1 and Table 2). Tumor size ranged from 0.5 cm to 12 cm with a median size of 3 cm. Patient cohort and clinical characteristics are summarized in Table 1.

**Table 2:** Summary of cytogenetic and molecular findings

Total # of cases	Patients	Karyotype *n* = 28	*BRAF*^V600E^ *n* = 24 (%) Mutation Wild Type		FISH for t(9;15) *n* = 20 (%)
	28	6 (21%)	15 (62%)	9 (38%)	2 (10%)
> 30 years	20	3	13	3	0
< 30 years	7	3	2	5	2 (100%)
Unknown	1	0	0	1	
Female (median age in years)	17 (52)	3	8	5	1
Male (median age in years)	10 (53.5)	3	7	3	1
Unknown	1	0	0	1	1

Out of 28 cases studied, cytogenetic analysis was available in only six (21%) cases, Table 2. Three (50%) cases out of 6 exhibited chromosomal aberration. All three patients were under the age of 30. Two cases exhibited translocation involving chromosome 9 (9p24) and chromosome 15 (15q24), or t (9;15)(p24;q24) [11, 13]. To rule out constitutional abnormality in these two patients, peripheral blood samples revealed normal karyotypes [11, 13]. One additional case that was translocation positive exhibited a translocation involving chromosome 6 (6q26) and chromosome 22 (q11.2) with a karyotype of 46,XX,t (6;22)(q26;q11.2). For this case, it is unknown if any genes are affected and if constitutional chromosome analysis was performed to rule out constitutional abnormality. The remaining three tumor cases demonstrated normal karyotypes of 46,XX or 46,XY and were over the age of 30. There were no differences noted among genders. Cytogenetic results are further summarized in Table 2.

The focus of the study was to determine the frequency of t (9;15) or *KANK1-NTRK3* gene fusion using FISH, Fig. 1, C and D. Cases 1 through 9 were tested using FISH probes RP11-130C19 on 9p24.3 (green signals) and RP11-62D2 on 15q25.3 (orange signals) from BlueGnome (Illumina, Cambridge, United Kingdom). Due to unavailability of previously used probes from BlueGnome, the following replacement probes were purchased from Empire Genomics (Buffalo, NY, USA): RP11-1107A23 on 9p24.3 (green signals) and RP11-608H9 on 15q25.3 (orange signals). A clinical cytogeneticist picked the best replacement probes based on available data from the vendor as well as University of California Santa Cruz (UCSC) Genome Browser (Genome Build 38), making sure probes covered the genes of interest. FISH analysis of the remaining cases (10 through 28) was carried out using the Empire Genomics probes. Clear signals and reportable FISH results were obtained in 20 (71%) cases. Of the 20 tumors with FISH results, two tumors (10%) were positive for *KANK1-NTRK3* fusion (Fig. 1, D). These two cases were also found to harbor the same translocation t (9;15)(p24;q24) by conventional cytogenetics analysis [11, 13]. Thus, concordance between FISH testing and chromosome analysis evaluation for the detection of the rearrangement and involvement of *KANK1-NTRK3* genes was 100% (2/2 cases). The remaining 18 (90%) tumors were negative for *KANK1-NTRK3* fusion, Fig. 1d. FISH in eight tumors (29%) was technically unsuccessful due to lack of hybridization signals and therefore uninformative. This was most likely due to DNA degradation in archival material, a conclusion supported by the fact that many of the same cases were uninformative for *BRAF* by PCR. FISH results are summarized in Table 2.

Of the 28 cases studied, the *BRAF* mutational analysis was informative in 24 cases (86%), Fig. 1e and f. *BRAF* exon 15 analysis was wild type (WT) in 9/24 (38%) cases (Fig. 1e) and mutated in 15/24 (62%) of cases, (Fig. 1f). Patient cases exhibiting *BRAF*^V600E^ mutation 13 of 15 (87%) were over the age of 30. The patients exhibiting *BRAF* wild type results were predominantly (5 of 9; 56%) under the age of 30. The Fisher exact test statistic value is 0.026. The result is significant at *p* < .05.

**Fig. 3:**
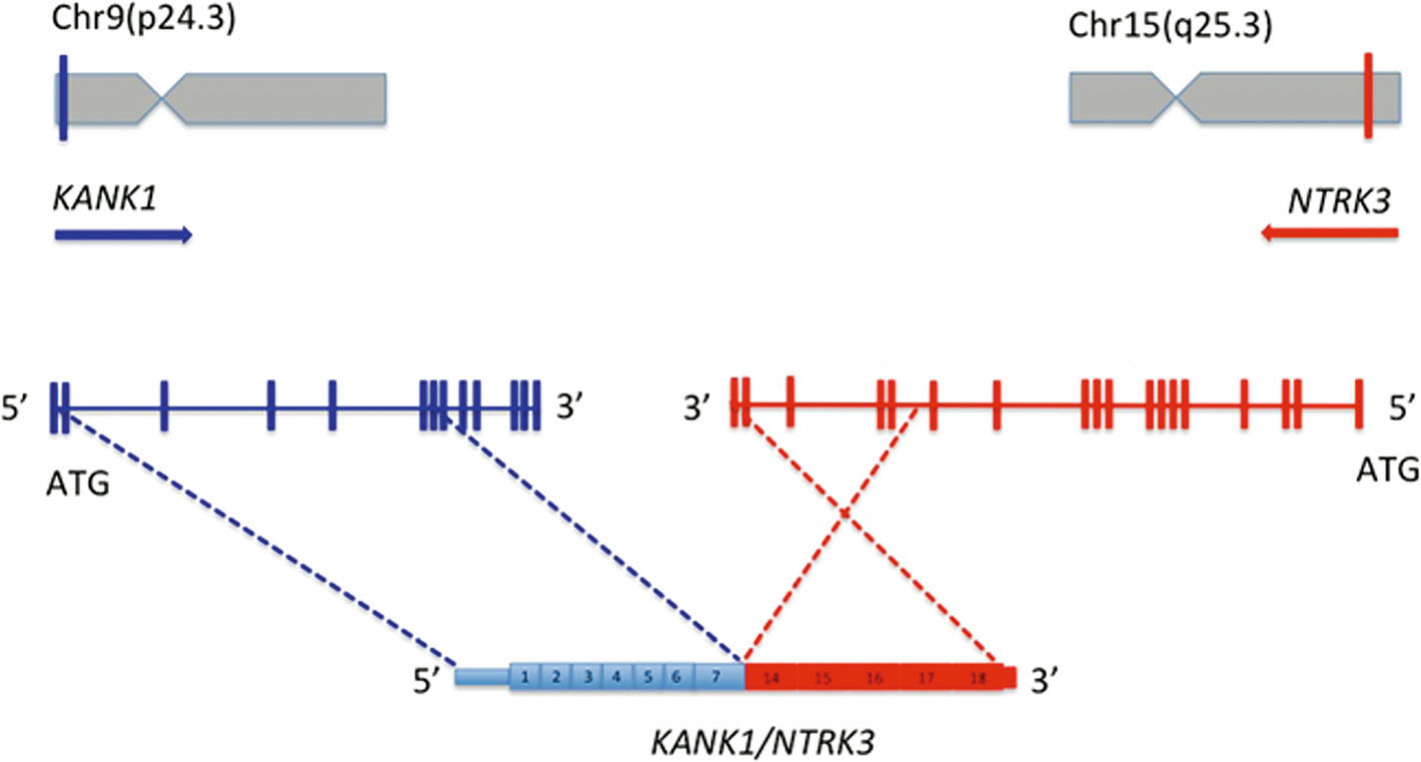
Schematic diagram of genomic location of *KANK1-NTRK3* fusion. Exons 1–7 of *KANK1* are shown in blue and exons 14–18 of *NTRK3* are shown in red

In the present study, the t (9;15) resulted in a *KANK1-NTRK3* fusion transcript in which the first seven exons of *KANK1* are fused to exon fourteen of the *NTRK3,* Fig. 3.

**Fig. 4:**
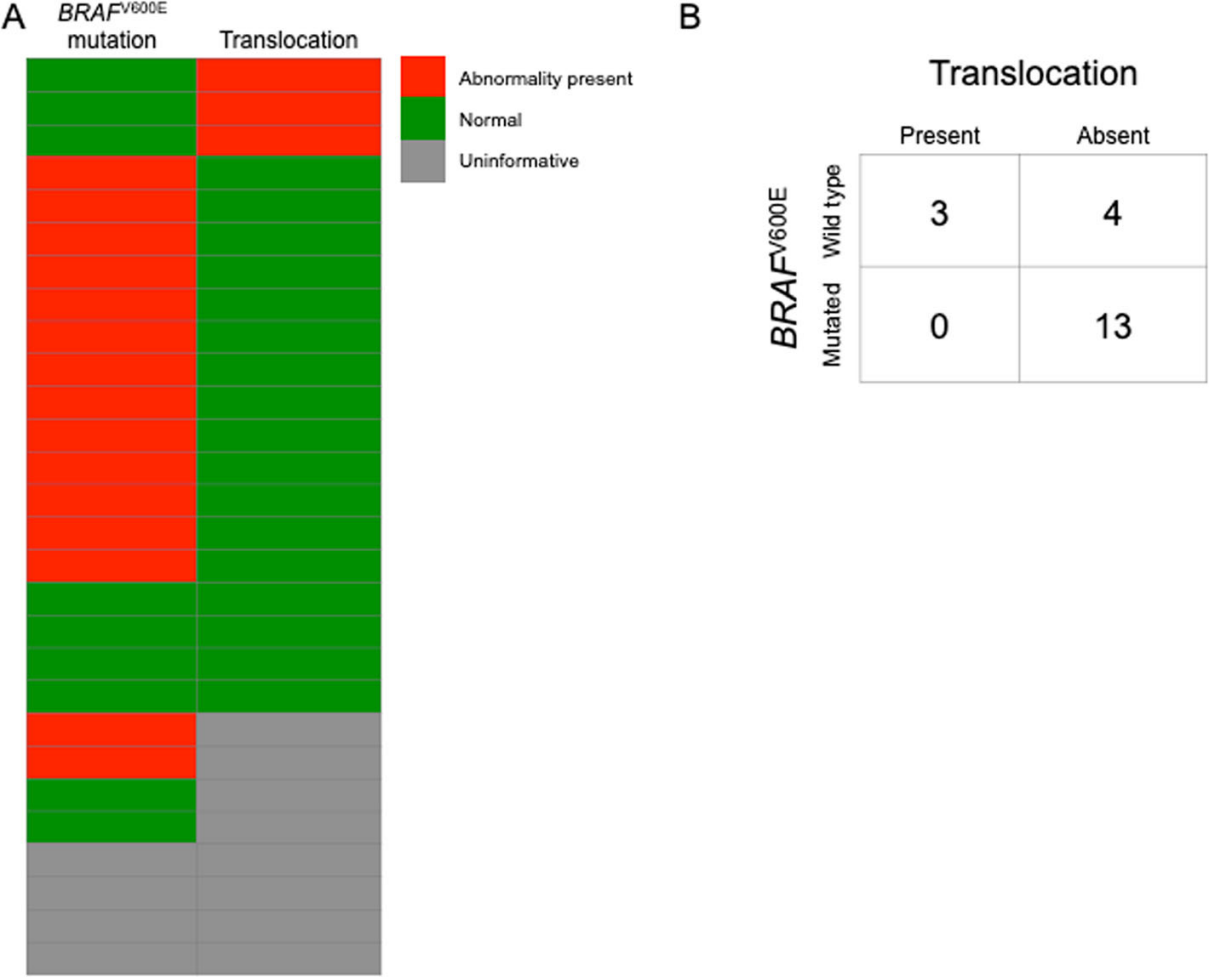
**a** Schematic representation of the *BRAF* status wild type (WT) is colored green and mutation is colored red. Translocation presence of either t(9;15) or t (6;22) is colored red and no translocation is colored green. Gray represents uninformative results. **b** Schematic representation of the results demonstrating the presence and the absence of translocation, and *BRAF*^V600E^ mutation or wild type, suggesting mutual exclusivity between *BRAF*^V600E^ and *KANK1-NTRK3* fusion

Out of the 9 cases lacking *BRAF* mutation, 3 cases (33%) exhibited chromosomal translocation. Four out of six *BRAF*^WT^ cases did not have cytogenetic results available, but showed normal FISH pattern for t (9;15). The remaining 2 cases were uninformative by FISH. Overall, 13 of 20 (65%) cases lacking t (9;15) harbored *BRAF*^V600E^ mutations. There were no cases demonstrating both the translocation and *BRAF*^V600E^ mutation, suggesting mutual exclusivity between *BRAF*^V600E^ and *KANK1-NTRK3* fusion, Fig. 4 (a and b).

## Discussion

Typically, straightforward cases of MA can be diagnosed solely based on histologic features. Challenging cases require additional diagnostic testing for distinction from malignant renal tumors. Cytogenetic analysis is labor-intensive, expensive, and generally not performed as part of routine clinical practice in classification of MAs; therefore, only a handful of previous studies have reported chromosomal aberrations in MAs. Catic et al. and Rakheja et al., observed balanced translocation t(9;15)(p24;q24) [11, 13], while Lerut et al., reported the presence of the dual balanced translocation, t(1,22)(q22;q13), and t(15;16)(q21;p13) [23]. However, several studies that have reported cytogenetic findings have reported normal karyotypes [23–30].

In this study, we confirm that a subset of biologically distinct MAs in younger patients (< 30 yrs. of age) exhibit aberrant chromosomal translocations and do not harbor *BRAF* mutations. The novel findings of our study are that the typical MAs which do not harbor *BRAF* mutations can demonstrate cytogenetic aberrations. Additional larger cohort studies are necessary to confirm and further elucidate the frequency of the cytogenetic aberrations found in this subset of MAs.

Unlike chromosome analysis data, FISH analysis and immunohistochemistry analyses on MA are more frequently performed and reported in the literature. Most reported FISH studies have been primarily focused on testing for trisomies of chromosome 7 and 17, and loss of Y chromosome. Brown et al., reported trisomies of chromosomes 7 and 17 and loss of Y chromosome in eight of 11 cases classified as MA [31]. However, their eight cases almost certainly represented examples of solid variant of PRCC, an entity that was described in the literature subsequent to their publication [32–34]. Recent studies strongly recommend utilization of FISH analysis to aid in differentiating those cases that deviate from the expected immunohistochemical staining pattern [3, 4].

Recent studies of MA have emphasized the importance of advanced molecular testing. Previous studies have demonstrated that the vast majority of MAs harbor *BRAF* ^V600E^ mutations, and that epithelial WTs lack *BRAF* ^V600E^ mutations [19, 20, 35]. *Choueiri* et al. published the largest series of MA demonstrating that 90% of MAs harbor a *BRAF* ^V600E^ mutation [14]. This was the first large study to shed light on the molecular underpinnings of MA and to investigate *BRAF*^V600E^ mutation in this indolent tumor. Additionally, they tested 129 non-MA renal neoplasms and detected *BRAF* ^V600E^ mutation in only one PRCC. Cytogenetic analysis of this PRCC case revealed the presence of trisomy of chromosomes 7 and 17. Padilha et al. and Choueiri et al. suggest that molecular *BRAF*^V600E^ mutation analysis is a valuable diagnostic tool in the differential diagnosis of this rare kidney tumor that may be diagnostically challenging [8, 14].

More recently, Chami et al. studied pediatric MA cases for *BRAF*^V600E^ mutations and found three out of four cases to be positive for *BRAF*^V600E^ mutation; 10 cases of pediatric renal cell carcinoma and 10 cases of Wilms’ tumor did not exhibit *BRAF*^V600E^ mutation [6]. To date, there are no cases reported in literature of Wilms’ tumor exhibiting *BRAF* mutations.

Similarly, Udager et al. evaluated eleven MA cases for *BRAF*^V600E^ mutations and found eight out of eleven cases to be positive for *BRAF*^V600E^ mutation. Of the three cases negative for *BRAF*^V600E^ mutation, two exhibited a novel *BRAF*^V600D^ mutation, of which one had a compound *BRAF*^V600D^ and *BRAF*^K601L^ mutation [4]. Overall, 90% of all published MA cases evaluated for *BRAF* mutations harbor *BRAF* exon 15 mutations. Most recent published study by Chan et al. examined the genetic profiles using next-generation sequencing on eleven conventional MAs and revelated all eleven cases harboring *BRAF* ^V600E^ mutations [35].

Our assay, which is capable of detecting 28 different *BRAF* mutations including *BRAF*^V600D^ and *BRAF*^K601L^ only detected *BRAF*^V600E^ mutations. Our results differ significantly from those in the literature in the overall lower frequency of *BRAF* mutations (62% vs. 90%). This may be due to the fact that in our study 25% of our patients were under the age of 30. We observed a possible trend towards patients younger than 30 years-old showing *BRAF*^WT^ tumors. This data suggests that MA in younger patients may be genetically distinct from its counterpart in older patients, but a larger patient cohort is needed to confirm this observation.

Lastly, we compared *BRAF*^V600E^ mutation analysis results with FISH for *KANK1-NTRK3* gene fusion. Of the nine cases that exhibited *BRAF*^WT^, 3 cases were those that demonstrated chromosomal aberrations by conventional karyotyping. Of the three cases that showed chromosomal translocations, two were positive for t (9;15) and *KANK1-NTRK3* gene fusion by FISH. The remaining six *BRAF*^WT^ cases did not have conventional cytogenetic analyses. Four of these six cases were negative for *KANK1-NTRK3* fusion by FISH, and 2 cases were uninformative. Overall, 13 of 20 (65%) cases lacking t (9;15) harbored *BRAF* mutations. There were no cases with both t (9;15) and *BRAF*^V600E^ mutation suggesting exclusivity between *BRAF*^V600E^ and t (9;15) and that the latter may be the genetic event behind a subset of *BRAF*^WT^ MAs.

A significant limitation of our study is the retrospective nature of case series with inability to test the BRAF-wild type cohort for additional mutations. Unfortunately, many cases had very little or no additional sample material to perform NTRK3, pan-Trk and BRAF immunohistochemistry testing.

## Conclusion

In conclusion, we report *KANK1-NTRK3* fusion without *BRAF*^V600E^ mutation in two MA cases. This finding supports the initial suggestion that *KANK1-NTRK3* is the pathogenetically significant fusion transcript in tumors carrying a t (9,15)(p24;q24) and lacking *BRAF*^V600E^ mutation in younger patient cohort. In this study, we have provided additional evidence that metanephric adenomas have relatively noncomplex karyotypes and have distinctive cytogenetic profiles. The cytogenetic profile can be useful in resolving a differential diagnosis of metanephric adenoma.

Classic histopathological features of MA coupled with a documented *BRAF*^V600E^ mutation are diagnostic of MA; however, we and others have demonstrated that the absence of *BRAF*^V600E^ mutation does not exclude a diagnosis of MA. For those case lacking *BRAF* mutations, alternative testing such as FISH analysis for *KANK1-NTRK3* fusion and/or cytogenetic chromosome analysis to look for t(9;15)(p24;q24) may be warranted.

## Data Availability

The data generated and/or analyzed during the current study is available from the corresponding author on reasonable request.

## Abbreviations

MA: Metanephric adenoma
FFPE: Formalin fixed paraffin-embedded
H&E: Hematoxylin and eosin
WHO: World Health Organization
PRCC: Papillary renal cell carcinoma
WT: Wilms’ tumor
ASI: Applied Spectral Imaging
ISCN: International System Committee for Human Cytogenomic Nomenclature
PCR: Real time polymerase chain reaction
CLIA: Clinical Laboratory Improvements Amendments
